# Identification of TRIM25 as a Negative Regulator of Caspase-2 Expression Reveals a Novel Target for Sensitizing Colon Carcinoma Cells to Intrinsic Apoptosis

**DOI:** 10.3390/cells8121622

**Published:** 2019-12-12

**Authors:** Usman Nasrullah, Kristina Haeussler, Abhiruchi Biyanee, Ilka Wittig, Josef Pfeilschifter, Wolfgang Eberhardt

**Affiliations:** 1 Institute of General Pharmacology and Toxicology, University Hospital, Goethe University Frankfurt am Main, D-60590 Frankfurt, Germany; nasrullah@em.uni-frankfurt.de (U.N.); haeussler@med.uni-frankfurt.de (K.H.); abhiruchi.biyanee@uni-muenster.de (A.B.); pfeilschifter@em.uni-frankfurt.de (J.P.); 2 Institute for Biochemistry, Westfälische Wilhelms-University Münster, D-48149 Münster, Germany; 3 Functional Proteomics, Medical School, Goethe University Frankfurt am Main, D-60590 Frankfurt, Germany; wittig@med.uni-frankfurt.de

**Keywords:** caspase-2, chemotherapy resistance, colon carcinoma cells, intrinsic apoptosis, TRIM25

## Abstract

Colorectal cancer (CRC) is one of the most common cancers that is characterized by a high mortality due to the strong metastatic potential of the primary tumor and the high rate of therapy resistance. Hereby, evasion of apoptosis is the primary underlying cause of reduced sensitivity of tumor cells to chemo- and radiotherapy. Using RNA affinity chromatography, we identified the tripartite motif-containing protein 25 (TRIM25) as a bona fide caspase-2 mRNA-binding protein in colon carcinoma cells. Loss-of-function and gain-of-function approaches revealed that TRIM25 attenuates the protein levels of caspase-2 without significantly affecting caspase-2 mRNA levels. In addition, experiments with cycloheximide revealed that TRIM25 does not affect the protein stability of caspase-2. Furthermore, silencing of TRIM25 induced a significant redistribution of caspase-2 transcripts from RNP particles to translational active polysomes, indicating that TRIM25 negatively interferes with caspase-2 translation. Functionally, the elevation in caspase-2 upon TRIM25 depletion significantly increased the sensitivity of colorectal cells to drug-induced intrinsic apoptosis as implicated by increased caspase-3 cleavage and cytochrome c release. Importantly, the apoptosis-sensitizing effects by transient TRIM25 knockdown were rescued by concomitant silencing of caspase-2, demonstrating a critical role of caspase-2. Inhibition of caspase-2 by TRIM25 implies a survival mechanism that critically contributes to chemotherapeutic drug resistance in CRC.

## 1. Introduction

The occurrence of drug resistance is a major obstacle that frequently limits the therapeutic benefit of cancer therapeutics. For this reason, the implementation of novel therapies that resensitize tumor cells towards current tumor therapies is urgently needed. In addition to disturbances in drug–target interactions leading to an increased drug efflux due to elevated expression of efflux transport proteins, e.g., the P-glycoproteins, defects in apoptosis programs represent further important reasons for therapy resistance (for a review, see [1,2]). Mechanistically, the reduced sensitivity towards apoptotic insults in tumor cells is achieved by the enhanced activation of cell survival pathways either due to mutations in tumor suppressor genes and/or as a consequence of impaired transcriptional or posttranscriptional control of apoptosis regulatory factors. A prominent example is the overexpression of members of the inhibitor of apoptosis (IAP) protein family, including the X-linked IAP (XIAP) and survivin, which is closely associated with a resistant phenotype of human malignancies, including advanced colorectal cancer [3,4]. As a consequence, therapy-resistant tumor cells are characterized by impaired activation of caspases, a family of cysteine-aspartate proteases that mediate the proteolytic processing of diverse downstream substrates in response to disruptive insults (for a review, see [2,5]). In addition to elevations in IAP proteins, we previously identified direct inhibition of caspase-2 translation by the ubiquitous mRNA-binding protein human antigen R (HuR) as a novel path of therapy resistance in colon carcinoma cells [6,7,8] (for a review, see [9]). In contrast to other members of the caspase family, the defined role of caspase-2 in apoptosis is still debated. Regardless, by acting as an initiator caspase, caspase-2 plays a critical role in the execution phase of apoptosis in response to DNA damage, genotoxic drugs, or mitotic catastrophe (for a review, see [10,11,12]).

In addition, recent data demonstrated that caspase-2 is critically involved in the control of various nonapoptotic functions, including tumor suppression [13,14,15,16,17], genomic stability [18,19], oxidative stress [20], and autophagy [21]. The pathophysiological relevance of caspase-2 is underscored by data from animal tumor models demonstrating that the loss of caspase-2 critically contributes to tumor development [13,14,15]. Correspondingly, diminished caspase-2 levels have been observed in different human carcinomas [22,23,24]. Although many studies have elucidated the different modes of enzymatic activation of caspase-2, less attention has focused on the control of caspase-2 expression.

In this study, using RNA affinity chromatography, we searched for RNA-binding proteins potentially involved in the regulation of caspase-2 translation. Thereby, we identified the tri-partite motif-containing protein (TRIM) 25, synonymously denoted as estrogen responsive finger protein (Efp), as a novel binding protein of caspase-2 mRNA. Originally, TRIM25 was described as an E3 ligase that mediates lysine (K) 63-linked polyubiquitination of the viral RNA sensor retinoic acid-inducible gene 1 (RIG-1), crucially involved in interferon signaling (for a review, see [25]). In addition to an antiviral cell response, evidence is accumulating that TRIM25 can exert important tumorigenic functions, e.g., control metastatic gene signatures at the transcriptional and post-transcriptional level [26]. Accordingly, an overexpression of TRIM25 has been demonstrated in many human cancers and correlates with a poor prognosis of patients (for a review, see [27]). Interestingly, recent data demonstrated a direct interconnection between the regulation of mRNA functions and the ubiquitin proteasome system. Although TRIM25 lacks any typical RNA binding domains (RBDs), previous studies showed that it can influence the processing and stability of bound mRNA (reviewed by [25]). In the present study, we unveil a novel mRNA modulatory function of TRIM25, which is critically involved in the negative regulation of caspase-2 in different colon carcinoma cell lines. Furthermore, our data implicate that inhibition of caspase-2 by TRIM25 protects tumor cells from chemotherapeutic drug-induced apoptosis and may constitute a novel mechanism of drug resistance in human colorectal carcinoma cells.

## 2. Materials and Methods

### 2.1. Reagents and Antibodies

Doxorubicin was purchased from Biotrend Chemicals (Cologne, Germany). Actinomycin D and rapamycin were obtained from Calbiochem (Schwalbach, Germany) and Merck KGaA (Darmstadt, Germany). The caspase-2-specific chemical inhibitor (Z-VDVAD-FMK) was from R & D Systems (Wiesbaden, Germany). Antibodies used in this study included the following: Anti-TRIM25 (#115737) from Abcam (Berlin, Germany) and (sc-166926) from Santa Cruz (Heidelberg, Germany), respectively. Anti-caspase-2 (#611022) from BD Biosciences (Heidelberg, Germany), anti-caspase-3 (#9662), anti-phospho-S6 ribosomal protein (Ser240/244, #2215), and anti-cytochrome c (#4272) from Cell Signaling (Frankfurt, Germany), anti-β-actin (#A2228) were from Sigma-Aldrich (Merck KGaA, Darmstadt, Germany). Secondary Horseradish Peroxidase HRP-linked antibodies sc-2054 and sc-20559 were obtained from Santa Cruz and a Cy5-coupled goat anti-mouse antibody was derived from Life Technologies (Darmstadt, Germany). Hyperfilm and the ECL system were purchased from GE Healthcare (Freiburg, Germany). Go-Taq polymerase was from Promega (Mannheim, Germany). Medium and supplements as well as modifying enzymes were from Invitrogen (Karlsruhe, Germany).

### 2.2. Cell Culture

The colon carcinoma cell lines DLD-1 bearing a heterozygous mutation in p53 were derived from the German Collection of Microorganisms and Cell Cultures (Braunschweig, Germany). The p53 wild-type cell line RKO and HEK293 cells were received from LGC-Promochem (Wiesbaden, Germany). Cells were maintained in Dulbecco′s modified eagle′s medium with 10% heat-inactivated fetal calf serum and a combination of 100 U/mL penicillin and 100 µg/mL streptomycin (Merck KGa, Frankfurt am Main, Germany).

### 2.3. RNA Interference

Subconfluent cells were transiently transfected with siRNAs by employing the Oligofectamine reagent from Invitrogen (Karlsruhe, Germany) according to the manufacturer′s instructions.

The transient knockdown of genes was achieved by transfection of siRNA-duplexes from 50 nM validated siRNAs from Thermo Scientific against human TRIM25 (ID#15204, “siTRIM25#1”), or alternatively, a mixture of FlexiTube siRNAs for human TRIM25 (SI0000072170, SI0000072163, SI0000072156, and SI0000072149, “siTRIM25#2”) from Qiagen (Hilden, Germany) or the same amounts of FlexiTube siRNA-duplexes for caspase-2 (SI00299551, “siCasp-2”) from Qiagen (Hilden, Germany). For double siRNA transfections with siTRIM25#1 and siCasp-2, each siRNA was employed at a concentration of 25 nM while a non-targeting siRNA (siCtrl, #D001206-13) from Dharmacon (St. Leon-Rot, Germany) was used as a control. 

### 2.4. Transient Overexpression of TRIM25

Subconfluent RKO cells were grown on 60-mm dishes and transiently transfected with 6 µg of pFLAG-CMV-TRIM25 (pFLAG-CMV2-EFP from Addgene), or alternatively, with the same amount of empty vector (pFLAG-CMV2) by using the Lipofectamine 2000 reagent (Thermo Scientific, Dreieich, Germany) by following the instructions of the manufacturer. Routinely, 48 h after transfection, the cells were used for further applications.

### 2.5. Western Blot Analysis

Cells were lysed by using a method described previously [6]. In brief, the cells were lysed in cold lysis buffer (137 mM NaCl, 20 mM Tris-HCl pH 8.0, 5 mM EDTA pH 8.0, 10% glycerol, and 1% Triton X-100) supplemented with a protease inhibitor mix from Roche (Mannheim, Germany). Snap-frozen lysates were subsequently subjected to five freeze-thaw cycles by using liquid nitrogen. Total cell lysates containing 20 to 30 µg of protein were prepared in SDS sample buffer and resolved by 12% or 15% SDS-PAGE and transferred for immunodetection onto nitrocellulose membranes by using specific primary antibodies and the appropriate secondary antibodies. Thereafter, signals were visualized with chemiluminescence using an ECL system from PerkinElmer (Rodgau, Germany). To confirm equal loading of protein amounts, blots were probed with β-actin antibodies.

### 2.6. Mitochondrial Cytochrome C Release

Apoptosis-induced release of cytochrome c from the mitochondrial intermembrane space into the cytoplasm [28] was monitored by cellular fractionation as described previously [8]. Thereafter, cytosolic cytochrome c levels were analyzed by Western blot analysis using specific antibodies.

### 2.7. qRT-PCR-Analysis

Total RNA was extracted from whole cells by using the Tri reagent (Merck KGaA). First strand cDNA from equal amounts of RNA was synthesized by using random hexamer primer and the RevertAid First Strand cDNA synthesis kit (Thermo Fisher Scientific, Darmstadt, Germany). Two-step real-time PCR was performed using a Taqman (ABI 7700) from Applied Biosystems. The mRNA levels for human TRIM25, caspase-2, and glyceraldehyde 3-phosphate dehydrogenase (GAPDH) were determined by using a protocol according to the ‘hot start′ real-time PCR procedure with gene-specific TaqMan probes (TRIM25: #Hs01116121_m1, Casp-2: #Hs00892481_m1 and GAPDH: #4310884E) from Thermo Scientific. (Cycle Threshold) ct values were normalized to the ct values of GAPDH mRNA within the same sample and quantified by using the 2−ΔΔCT method.

### 2.8. Ribonucleoprotein- (RNP) IP RT-PCR Assay

Endogenous TRIM25-mRNA complexes were precipitated by using RNP-IP-qRT-PCR analysis as described previously [29].

Briefly, cells were treated with ice-cold lysis buffer complemented with 40 U/mL RNasin. For immunoprecipitation (IP) of endogenous TRIM25, total cell lysates (500 µg) were incubated overnight at 4 °C either with 2 µg of a monoclonal anti-TRIM25 antibody (#sc-166926) diluted in lysis buffer containing 5% fetal calf serum. Instead of anti-TRIM25 antibodies, the same amount of mouse (immunoglobulin G) IgG was used in control IP reactions. Routinely, the cell lysates were precleared for 1 h at 4 °C with protein G-Sepharose 4 Fast Flow (GE Healthcare, Freiburg, Germany) before antibodies were added. Subsequently, fresh protein G-Sepharose beads were added and incubated at 4 °C for another 2 h with continuous rotation. After centrifugation for 2 min at 3000× *g*, precipitates were washed three times with low-salt buffer (50 mM Tris-HCl pH 7.5, 150 mM NaCl, 0.2% Triton X-100, 2 mM EDTA, 2 mM EGTA, 0.1% SDS, 40 U/mL RNasin) and three times with high-salt buffer (50 mM Tris-HCl pH 7.5, 500 mM NaCl, 0.2% Triton X-100, 2 mM EDTA, 2 mM EGTA, 0.1% SDS, 40 U/mL RNasin), respectively. Thereafter, the precipitated RNA was extracted by using the Tri reagent. TRIM25-bound transcripts were reverse transcribed (RT) using a random hexamer primer and the RevertAid First Strand cDNA synthesis kit (Thermo Fisher Scientific, Darmstadt, Germany) and analyzed by quantitative RT-PCR. The following primer sets were used: Caspase-2 fwd.: 5′-ACA GGG GAC GCA GGA TAT TGG GA-3′, Caspase-2 rev.: 5′-GGT GGC CTT GCT TGG TCT CCC T-3′; GAPDH fwd.: 5′-CAC CAT CTT CCA GGA GCG AG, GAPDH rev.: 5′-GCA GGA GGC ATT GCT GAT -3′. The PCR products were separated on a 1% agarose gel containing GelRed from Biotium (Biotrend, Cologne, Germany).

In addition, semiquantitative RT-PCR was performed by using Go-Taq hot start polymerase (Promega, Mannheim, Germany) and conditions described for quantitative PCR.

Normalization of input RNA was confirmed by RT reaction of total cellular RNA isolated from 10% of cell extract as was used for IP and subsequent assessment of GAPDH levels. Similarly, mRNA complexes associated with ectopically expressed Flag-tagged TRIM25 were precipitated by incubation of anti-Flag-M2 magnetic beads with 500 µg of total cell lysates from RKO cells overexpressing Flag-tagged cDNA constructs. To reduce unspecific binding of the tag to RNA, cell lysates were precleared with 1 µg of transfer RNA for 1 h at 4 °C with continuous rotation before the addition of the magnetic beads. The protocol used for RNA isolation was similar as described ahead.

### 2.9. RNA Affinity Chromatography

RNA affinity chromatography (Biotin pull down) was performed as described in [7]. In brief, 10 µg of BamHI-linearized plasmid bearing pCR2.1-5′-UTR-caspase-2 was used as an RNA sense probe (based on NM_032982) with the help of a RiboMax-Large Scale RNA Production System-T7 from Promega. Subsequently, in vitro transcribed RNA was biotinylated at the 5′-end with biotin-maleimide (Linaris, Dossenheim, Germany) by use of an End Taq Labeling Kit from Vector Laboratories (Biozol, Eiching, Germany). In total, 20 µg of biotin-labeled transcript was mixed with streptavidin-conjugated agarose beads and incubation buffer (10 mM Tris-HCl, pH 7.5; 150 mM KCl; 1.5 mM MgCl_2_; 0.5 mM DTT supplemented with 40 U/mL RNasin) at 4 °C for 2 h under rotation. Thereafter, 300 µg of total cell extract were incubated with the beads for 1 h at 4 °C with continuous rotation. Beads were washed several times with incubation buffer before being resuspended with 20 µL of Laemmli buffer and heated at 95 °C for 10 min. Eluted pull-down material was analyzed by Western blot analysis using a TRIM25-specific antibody and equal input material was confirmed by Western blotting with the same antibody used for the detection of pull-down material.

### 2.10. Liquid Chromatography/Mass Spectrometry (LC/MS)

For the identification of novel 5′UTR-caspase-2-binding proteins, RNA affinity chromatography was performed as described above, but in contrast to resuspending the protein bound beads with Laemmli buffer, they were incubated with 50 µL of 8 M urea, 50 mM Tris/HCl, pH 8.5, 10 mM DTT, and incubated at 22 °C for 30 min. Reduced thiols were alkylated with 40 mM chloroacetamide and samples were diluted with 25 mM Tris/HCl, pH 8.5, 10% acetonitrile to obtain a final urea concentration of 2 M. After digestion with trypsin, the remaining peptides were analyzed by using LC/MS on a Q Exactive Plus from Thermo Scientific™ and an ultra-high-performance liquid chromatography unit (Thermo Scientific Dionex Ultimate 3000). For peptide identification, raw files were directly searched against the Uniprot human reference proteome using the Mascot 2.2. search engine.

### 2.11. Separation of Polysomes from Translational Inactive RNP Granules

Separation of polysomes from RNP granules was achieved as described previously [30]. Briefly, subconfluent cells from at least three 6-cm dishes were scrapped in ice-cold PBS/0.02% EDTA buffer and collected by centrifugation for 5 min at 3000× *g* at 4 °C before the cell pellets were resuspended with ice-cold lysis buffer (137 mM NaCl, 20 mM Tris-HCl pH 8.0, 5 mM EDTA pH 8.0, 10% glycerol, and 1% Triton X-100, 100 U/mL RNasin) and protease inhibitor mix (Roche, Mannheim, Germany) followed by centrifugation at 10,000× *g* or 15 min at 4 °C. Supernatants were pooled and equal protein amounts (500 to 1000 µg) were loaded on a sucrose cushion (1 M). Polysomes were isolated by centrifugation at 100,000× *g* for 2 h at 4 °C without a brake using a fixed angle rotor (in a Beckmann ultracentrifuge and polysomal pellets dissolved in ice-cold polysome extraction buffer (PEB) buffer (10 mM Tris-HCl, 100 mM NaCl, 10 mM EDTA, 1% SDS, pH 7.4, 100 U/mL RNasin)). For isolation of postpolysomal RNP fractions, the sucrose-containing supernatants were centrifuged a second time at 300,000× *g* for 3 h at 4 °C and pellets with RNPs dissolved in PEB buffer. RNA from both fractions was precipitated overnight with 5 M LiCl and absolute ethanol. The precipitated RNA was further purified by using the Nucleo Spin RNA Kit (Machery-Nagel, Düren, Germany) following the manufacturer′s instructions. After cDNA synthesis, individual mRNA contents were measured by semi-quantitative RT-PCR as described before.

### 2.12. Confocal Microscopy

Staining of intracellular TRIM25 was performed by a confocal microscopy as described [31]. Colon carcinoma cells were seeded on cover glasses in 12-well plates (neoLab, Heidelberg, Germany) before chemotherapeutic drugs were administered. Thereafter, cells were exposed to 4% paraformaldehyde plus 0.25% Triton X-100 (AppliChem, Darmstadt, Germany) in PBS for 15 min for fixation and permeabilization. After incubation in blocking solution (5% BSA in PBS), a monoclonal anti-TRIM25 antibody was added for 1 h at room temperature. Thereafter, cells were washed several times with PBS before being incubated with a Cy5-conjugated anti-mouse antibody. Nuclei were counterstained with 4′,6-diamidino-2-phenylindole (DAPI) (Life Technologies) for 2 min and finally washed with PBS. Stained cells were finally monitored by using an LSM510 inverted laser scanning microscope from Zeiss (Göttingen, Germany). Image analysis was performed with the help of ZEN2009 Light Edition software from Zeiss. 

### 2.13. Statistical Analysis

Most experiments shown were performed at least three times. For proof of the statistical relevance, the unpaired two-tailed *t*-test was used. *p* values ≤ 0.05 were considered as significant.

## 3. Results

### 3.1. Identification of TRIM25 as a Novel Caspase-2 mRNA-Binding Protein

**Figure 1:**
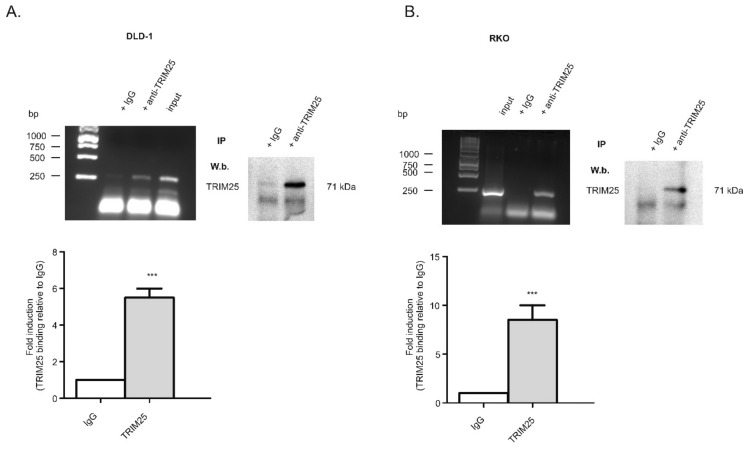
TRIM25 is a bona fide caspase-2 mRNA-binding protein in the human colon carcinoma cell line DLD-1 (**A**) and RKO (**B**). RNP-IP assays from total cell lysates of colon carcinoma cells were performed and followed by RT-PCR. TRIM25-bound mRNA was precipitated by the addition of monoclonal TRIM25-specific antibodies (anti-TRIM25) or, alternatively, the same amount of mouse IgG (IgG) as a negative control. RNA samples were analyzed by semiquantitative RT-PCR using primersets encompassing the coding region of caspase-2. The specific immunoprecipitation (IP) of TRIM25 in both cell lines was validated by Western blot analysis (W.b.) as shown on the right-hand side. Input levels of caspase-2 mRNA were monitored by RT-PCR (input). Graphs shown in the lower parts of the panels show means ± SD (*n* = 3) and depict the relative caspase-2 binding to TRIM25 (filled bars) in relation to IgG (open bars). *** *p* ≤ 0.001 vs. IgG.

Previously, we discovered a cell survival mechanism in colon carcinoma cells by which translation of the pro-apoptotic caspase-2 is constitutively repressed by the ubiquitous mRNA-binding protein, (human antigen R) HuR [6,8]. In order to identify further RNA-binding proteins that are critical for caspase-2 translation, we performed streptavidin-tethered RNA affinity chromatography in combination with mass spectrometry using total cell homogenates from untreated DLD-1 cells. Since the negative regulation of caspase-2 by HuR depends on the 5′untranslated region (5′UTR), for affinity purification, biotin-labelled in vitro-transcribed mRNAs encompassing either the 5′-UTR of caspase-2, or alternatively, the coding region (cdr) of caspase-2 were used as baits. Proteins that were bound to biotin-labelled RNAs were eluted and subsequently analyzed by mass spectrometry. Among various eukaryotic translation initiation factors and some well-known RNA-binding proteins, including HuR, we identified the tripartite motif-containing protein (TRIM) 25, synonymously denoted as estrogen-responsive finger protein (Efp), as a protein strongly associated with the 5′UTR but only with a weak affinity to the cdr of caspase-2 mRNA (Supplementary Table S1). Although results from the mass spectrometry indicated a relatively low caspase-2 mRNA-binding affinity, we chose this candidate because TRIM25 has previously been reported as a key determinant of breast cancer metastasis [26], suggesting that it could also exert a tumorigenic role in colon carcinoma. An RNA-specific binding of TRIM25 to caspase-2 mRNA in DLD-1 cells was validated by the RNP-IP RT-PCR assay (Figure 1A) and confirmed in the colorectal cancer cell line RKO (Figure 1B). Western blot analysis furthermore affirmed the specific binding of the antibody used for RNP-IP (right panels of Figure 1A,B). In addition, the specificity of TRIM25 binding is indicated by the inability of the control, IgG, to yield appropriate PCR amplicons (Figure 1A,B).

### 3.2. TRIM25 Negatively Interferes with Caspase-2 Translation

**Figure 2:**
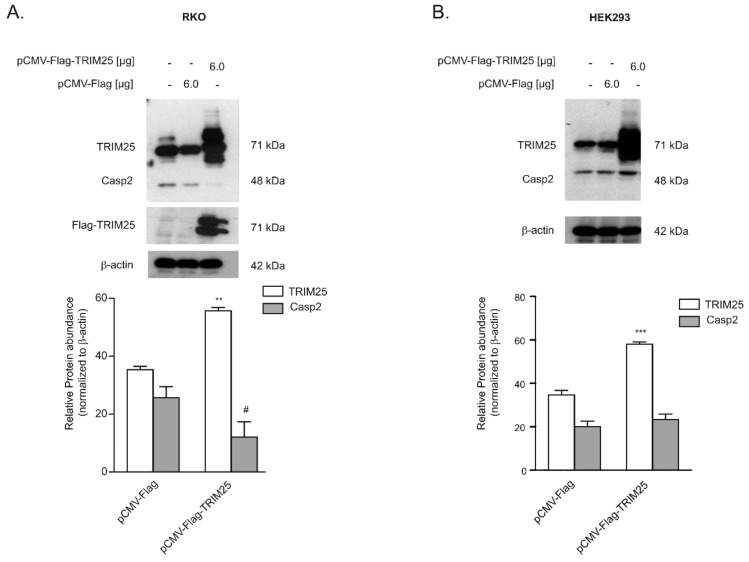
TRIM25 reduces abundance of caspase-2 protein in RKO cells but not in HEK cells. Subconfluent RKO (**A**) or HEK293 (**B**) cells mock transfected (-) or transfected with the indicated amount of Flag-tagged human TRIM25 (pCMV-Flag-TRIM25) or, alternatively, with the same amount of empty pCMV-Flag vector (pCMV-Flag). Then, 48 h after transfection, the amounts of endogenous and ectopic TRIM25 and caspase-2 were monitored by Western blot analysis and β-actin was used as a loading control. Graphs at the bottom show means ± SD (*n* = 3) and depict the relative abundance of TRIM25 (open bars) and caspase-2 (filled bars) protein in both transfected cell populations. ** *p* ≤ 0.005, *** *p* ≤ 0.001 (TRIM25), and # *p* ≤ 0.05 (Casp2), respectively, vs. empty vector transfected.

Next, we tested whether TRIM25 binding to caspase-2 mRNA would affect the translation of caspase-2 in a similar way as we previously reported for HuR [6]. To this end, we analyzed whether overexpression of TRIM25 would influence the abundance of caspase-2 protein. Due to the lower transfection efficiencies in DLD-1 cells, these experiments were performed in RKO cells. Transient transfection of Flag-tagged TRIM25 caused a robust overexpression of TRIM25 and was concomitant with a clear and significant reduction in caspase-2 levels. Interestingly, a lower migrating immunopositive band, which mainly appeared with ectopically expressed TRIM25 but also with endogenous TRIM25, was also detected by using an anti-Flag antibody, suggesting that it probably results from a post-translational modification of TRIM25. In contrast, an additional faster migrating band in the lysates from ectopically expressed cells was not recognized by the anti-Flag antibody, indicating that it may either represent an unspecific protein or results from a TRIM25 degradation product devoid of the Flag tag. Instead, transfection of the empty vector did not significantly affect the content of caspase-2 protein (Figure 2A). Notably, attenuation in caspase-2 levels upon ectopic TRIM25 expression was not observed in HEK cells, indicating that the inverse correlation of both proteins may reflect a cell type-specific phenomenon (Figure 2B).

**Figure 3:**
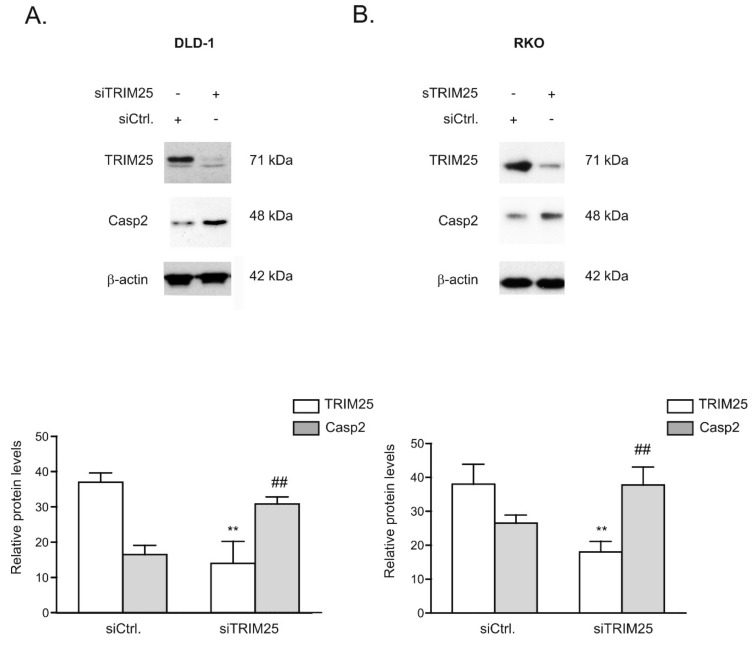
Silencing of TRIM25 increases caspase-2 protein levels in colon carcinoma cells. Subconfluent DLD-1 (**A**) or RKO (**B**) cells were transfected with control siRNA duplexes (siCtrl.) or with siRNA duplexes of TRIM25 (siTRIM25). Then, 48 h after transfection, cells were harvested and total protein lysates (20 µg) were subjected to SDS-PAGE. The abundance of TRIM25 and caspase-2 was analyzed by Western blot analysis and β-actin was used as a loading control. Graphs at the bottom show means ± SD (*n* = 3) and depict the relative protein levels of TRIM25 (open bars) and caspase-2 (filled bars) protein. ** *p* ≤ 0.005, (TRIM25) and ## *p* ≤ 0.005 (Casp2), respectively, vs. control siRNA-transfected (siCtrl.) cells.

Next, the inverse correlation between TRIM25 and caspase-2 proteins was tested by employing transient TRIM25 knockdown. Monitoring of the time-dependent effectiveness of TRIM25 knockdown (siTRIM25) revealed a strong and stable decrease in TRIM25 protein expression in both colon carcinoma cell lines tested, whereas transfection with a scrambled siRNA (siCtrl.) had no effects (Figure 3A,B, Figure S1A,B). Importantly, in both tested cell lines, the increase in caspase-2 levels upon TRIM25 knockdown was only transient and most obvious 48 h after siRNA transfection (Figure 3A,B, Figure S1A,B). Similar to the effects observed after knockdown of TRIM25, an inducible effect on caspase-2 levels, which was only transient, was previously observed upon HuR depletion, indicating the existence of compensatory mechanisms that counter-regulate the forced increase in caspase-2 in colon carcinoma cells [7]. For this reason, we continued further experiments with an siRNA-mediated approach rather than employing a stable knockdown of TRIM25. A similar TRIM25 knockdown-dependent rise in caspase-2 protein was observed with siRNA duplexes targeting another sequence of TRIM25 (Figure S1C,D), supporting that the induction of caspase-2 does not result from off-target effects. Furthermore, the increase in caspase-2 upon TRIM25 knockdown was only observed on the protein level but not on the mRNA level, independent of the colon carcinoma cell line that was assessed (Figure S2A,B). Importantly, the protein content of caspase-2 was strongly diminished after 72 h of cell culturing on Petri dishes probably because the synthesis and/or the stability of caspase-2 protein is negatively affected if cells are cultured for such long periods (Figure S1A,B).

**Figure 4:**
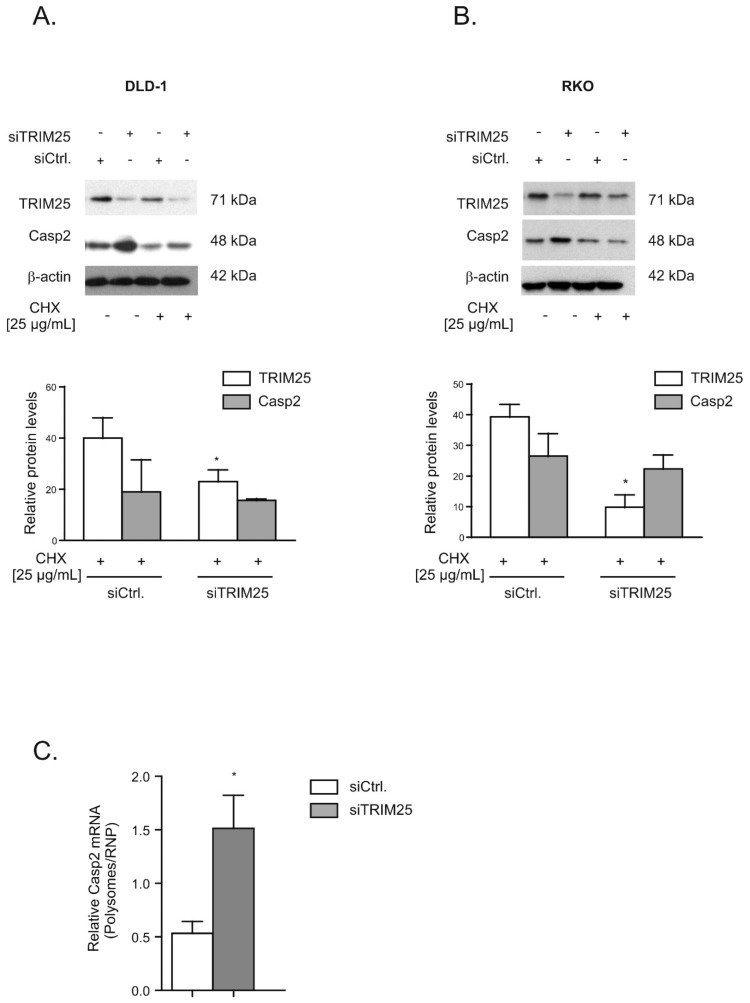
TRIM25 silencing-mediated increase in caspase-2 depends on de novo protein synthesis. Subconfluent DLD-1 (**A**) or RKO (**B**) cells were transfected with control siRNA duplexes (siCtrl.) or with siRNA duplexes of TRIM25 (siTRIM25) for 24 h before translation was blocked by the addition of cycloheximide (CHX, 25 µg/mL). After an additional 24 h, cells were harvested for total protein extraction. Total protein lysates (20 µg) were subjected to SDS-PAGE before the abundance of TRIM25 and caspase-2 was analyzed by Western blot analysis, with β-actin used as a loading control. Graphs at the bottom show means ± SD (*n* = 3) and depict the relative protein levels of TRIM25 (open bars) and caspase-2 (filled bars) protein at 24 h after CHX application. * *p* ≤ 0.05 (TRIM25) vs. control siRNA-transfected (siCtrl.) cells. (**C**). Subconfluent DLD-1 cells were transfected with control siRNA duplexes (siCtrl.) or with siRNA duplexes of TRIM25 (siTRIM25) for 48 h before fractions of translational inactive RNPs were separated from translational active polysomes by ultracentrifugation. The amount of caspase-2 mRNA was determined by qRT-PCR using primer pairs that were complementary and specific to the coding region of caspase-2. The graph shows means ± SD (*n* = 3) and depicts the amount of caspase-2 mRNA levels normalized to GAPDH mRNA in polysomes relative to those in RNP fractions from control siRNA- (open bars) and TRIM25 siRNA-transfected cells (filled bars). * *p* ≤ 0.05 (TRIM25) vs. control siRNA-transfected (siCtrl.) cells.

Together, these observations imply that the negative effects by TRIM25 on caspase-2 are not due to changes in caspase-2 mRNA levels but rely mainly on the modulation of caspase-2 protein content. To test whether the increase in caspase-2 upon TRIM25 knockdown is mainly due to an enhanced translation or, alternatively, would rely on increased protein stability, we monitored the time-dependent decay of caspase-2 in the presence or absence of cycloheximide (CHX), an inhibitor of eukaryotic translation (Figure S3). After the administration of CHX 24 h post transfection, we found that the TRIM25 silencing-dependent increase in caspase-2 was reduced in the presence of CHX, with a total loss in caspase-2 increase seen after an additional 24 h (Figure 4A). In contrast, the levels of TRIM25 were not affected by CHX, indicating that the TRIM25 protein has a long half-life. Similar results were obtained with RKO cells (Figure 4B). Together, these results indicate that the increase in caspase-2 protein in TRIM25 knockdown cells depends on de novo protein synthesis but does not result from elevated caspase-2 protein stability or changes in corresponding mRNA expression levels. This assumption was further verified by the results from polysomes/RNP fractionation experiments. Due to the low yield of input material from transfected cells, we used a protocol that crudely separates fractions derived from low- and high-molecular weight polysomes from fractions containing translational inactive RNPs. Quantification of caspase-2 mRNA levels in polysomes and nonpolysomal RNP fractions from both transfected cell populations (siCtrl. and siTRIM25) by quantitative RT-PCR revealed a significant increase in the relative polysome:RNP ratio of caspase-2 mRNA in siTRIM25-transfected cells, indicating increased caspase-2 translation when compared to control siRNA-transfected cells (Figure 4C).

### 3.3. Knockdown of TRIM25 Sensitizes Colon Carcinoma Cells to Drug-Induced Apoptosis

**Figure 5:**
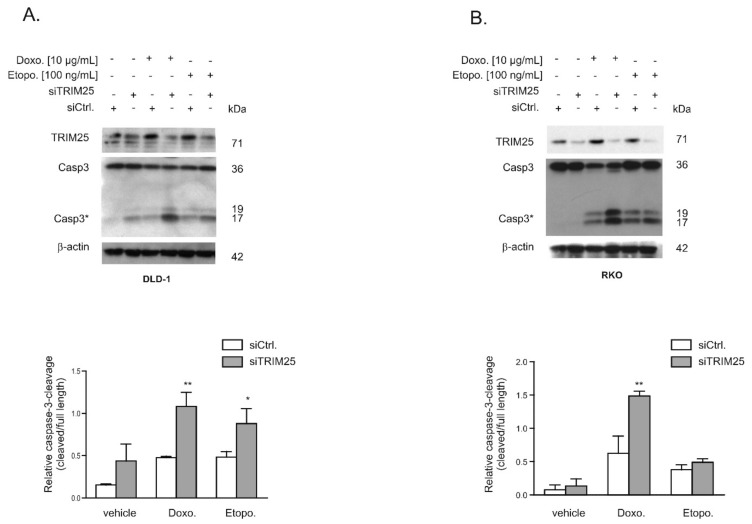
Knockdown of TRIM25 causes an increase in chemotherapeutic drug-induced intrinsic apoptosis in colon carcinoma cells. DLD-1 cells (**A**) or RKO cells (**B**) were either transfected with control siRNA (siCtrl.) or with siRNA duplexes against TRIM25 (siTRIM25) for 24 (**A**) or 48 (**B**) h before stimulation with doxorubicin (Doxo.) or etoposide (Etopo.) for a further 24 h. Thereafter, the cells were lysed for total protein extraction and caspase-3 cleavage and knockdown of TRIM25 were determined by Western blot, with β-actin used for equal loading. Graphs at the bottom summarize the densitometric analysis of cleaved caspase-3 (Casp3*) levels in relation to full-length caspase-3. Data show means ± SD (*n* = 3) * *p* ≤ 0.05, ** *p* ≤ 0.01 siTRIM25 (filled bars) vs. siCtrl. (open bars) transfected cells.

**Figure 6:**
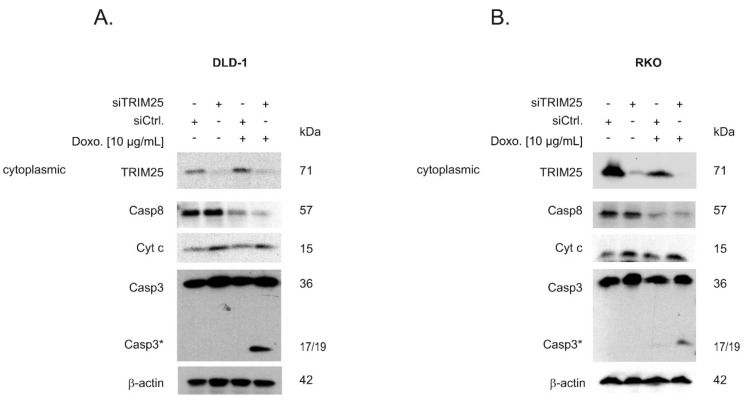
Silencing of TRIM25 via increased mitochondrial cytochrome c release leads to a sensitization of doxorubicin-induced caspase-3 cleavage. DLD-1 cells (**A**) or RKO cells (**B**) were transfected with control siRNA duplexes (siCtrl.) or with siRNA duplexes targeting TRIM25 (siTRIM25) for 48 h before being stimulated with the indicated doses of doxorubicin (Doxo.). After a further 24 h, cells were harvested for cytoplasmic cell lysates. The content of cytoplasmic cytochrome c (Cyt c), mature caspase-8, and cleavage of caspase-3 as well as the knockdown efficiency of TRIM25 was subsequently determined by Western blot using β-actin as a loading control. Data are representative of two independent experiments giving similar results.

Given the functional role of caspase-2 in DNA damage-induced cell death [7,8], we tested whether an increase in caspase-2 levels after knockdown of TRIM25 has a sensitizing effect on colorectal cancer cells towards drug-induced intrinsic apoptosis by monitoring cleavage of caspase-3 as the major effector caspase. In a first set of experiments, we tested two clinically established topoisomerase II inhibitors: Etoposide and the DNA intercalating anthracycline antibiotic, doxorubicin. To achieve maximal effects, the drugs were applied 24 to 48 h post-transfection, when caspase-2 levels peaked and caspase cleavage was measured after an additional 24 h (Figure 5A,B). Western blot analysis revealed a weak basal and a robust drug-induced processing of the effector caspase-3, which was significantly increased after knockdown of TRIM25 (Figure 5A). When testing RKO cells, we found a similar significant sensitizing effect by TRIM25 silencing only with doxorubicin but not with etoposide (Figure 5B). Next, we tested whether the increase in the cleavage of effector caspases by doxorubicin would correlate with an increase in cytochrome c release. Interestingly, results from cell fractionation and subsequent Western blot analysis showed that TRIM25 silencing caused a moderate increase in cytosolic cytochrome c levels, which were not further increased by doxorubicin (Figure 6A). In contrast, a cytoplasmic caspase-3 cleavage product at 17/19 kDa was only detected in siTRIM25-transfected DLD-1 cells that were treated with doxorubicin (Figure 6A). Notably, when monitoring caspase-8 cleavage, we observed a robust decrease in procaspase-8, an initiator caspase of receptor-dependent apoptosis, by doxorubicin, which is in line with previous studies showing an induction of FAS-mediated cell death by this chemotherapeutic agent [32]. Similar effects were observed in RKO cells (Figure 6B). Together, these data suggest that the increased sensitivity of colon carcinoma cells to doxorubicin-induced apoptosis by TRIM25 silencing results from convergent activation of mitochondrial apoptosis (via silencing of TRIM25) and drug-mediated activation of receptor-triggered apoptosis.

### 3.4. Caspase-2 is Critical for Sensitization by TRIM25 Silencing

**Figure 7:**
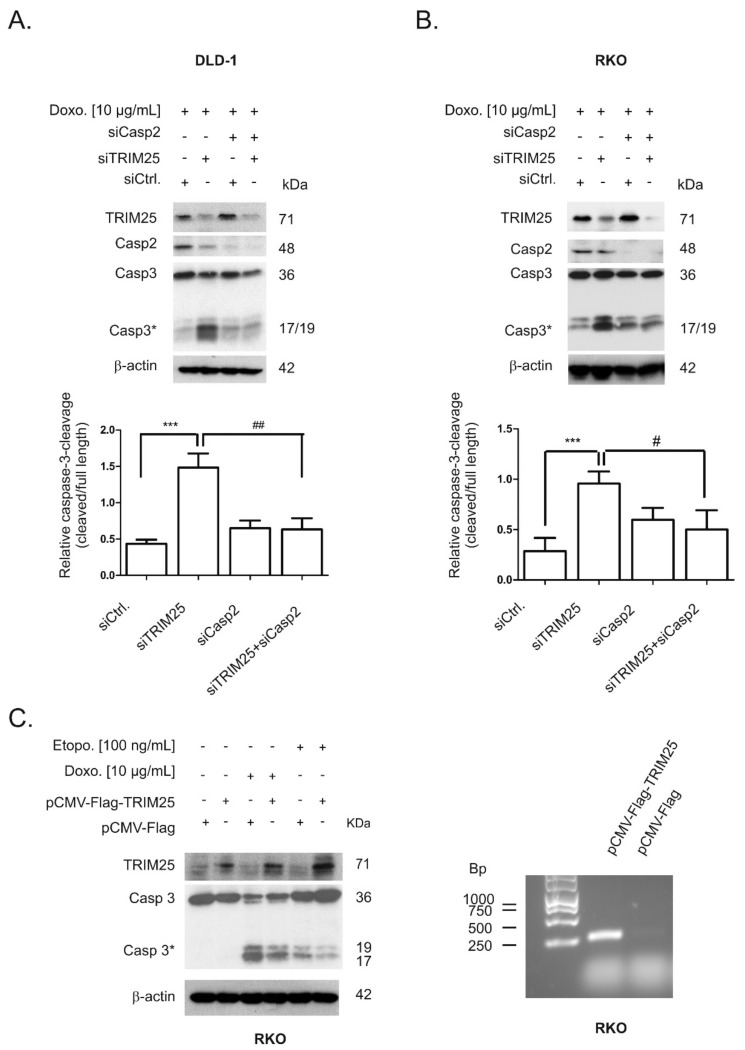
Silencing of caspase-2 prevents doxorubicin-induced apoptosis upon TRIM25 knockdown. DLD-1 (**A**) or RKO (**B**) cells were transfected with control siRNA (siCtrl.) or with siRNA duplexes either targeting caspase-2 (siCasp2) or TRIM25 (siTRIM25) or, alternatively, simultaneously transfected with TRIM25 plus caspase-2-specific siRNAs for 24 (**A**) or 48 (**B**) h before cells were exposed to doxorubicin for a further 24 h. Thereafter, cells were collected for total protein extraction and activation of caspase-3 cleavage and knockdown efficiency of TRIM25 or caspase-2 determined by Western blot using β-actin as a control for equal protein loading. Graphs summarize a densitometric analysis of cleaved caspase-3 (Casp3*) levels in relation to full-length caspase-3. Values show means ± SD (*n* = 3) *** *p* ≤ 0.005 siTRIM25 vs. siCtrl. and # *p* ≤ 0.05, ## *p* ≤ 0.01 siTRIM25/Casp2 vs. siTRIM25. (**C**, left panel). Subconfluent RKO cells were transfected for 48 h with 6 µg of Flag-tagged human TRIM25 (pCMV-Flag-Trim25) or, alternatively, with the same amount of empty pCMV-Flag vector (pCMV-Flag) before being treated with either the vehicle (-) or with the indicated chemotherapeutic drugs. After a further 24 h, cells were harvested for total protein extraction and the cleavage of caspase-3 as well as the overexpression of TRIM25 was determined by Western blot using β-actin as a loading control. (C, right panel). RNP-IP assays from total cell lysates of RKO cells expressing either Flag-tagged human TRIM25 (pCMV-Flag-TRIM25) or pCMV-Flag (pCMV-Flag). TRIM25-bound mRNA was isolated by the use of anti-Flag-M2 magnetic beads followed by RT-PCR. For PCR, primer pairs, complementary and specific to the coding region of caspase-2, were used and the length of specific PCR products determined by a DNA ladder. Data shown are representative of three independent experiments giving similar results.

Next, we tested the functional impact of caspase-2 on the increased cleavage of both effector caspases-3 by TRIM25 depletion by the additional silencing of caspase-2. As previously demonstrated, the transfection of two different sets of caspase-2-specific siRNA duplexes caused a similar reduction in caspase-2 levels in colon carcinoma cells [8]. Western blotting of total cell lysates confirmed a high knockdown efficiency of TRIM25 and caspase-2 in DLD-1 and RKO cells (Figure 7A,B). In full accordance with the results obtained with cytoplasmic fractions (Figure 6A,B), TRIM25 silencing significantly sensitized DLD-1 cells to caspase-3 cleavage by doxorubicin (Figure 7A). Similarly, the doxorubicin-induced processing of caspase-2 (indicated by a reduction in mature caspase-2) was enhanced after TRIM25 silencing (Figure 7A). As expected, the additional knockdown of caspase-2 did completely reverse the TRIM25 silencing and drug-induced increase in the processing of caspase-3 (Figure 7A). A caspase-2-dependent increase in doxorubicin-induced caspase-3 cleavage was also observed in RKO cells (Figure 7B). Reciprocally, ectopic expression of TRIM25 caused a reduction in drug-induced cleavage of caspase-3 (Figure 7C, left panel). Before, the specific interaction of Flag-tagged TRIM25 with caspase-2 mRNA was confirmed by RNP-IP RT-PCR assay (Figure 7C, right panel). Together, these results demonstrate that silencing of TRIM25 expression sensitizes colon carcinoma cells to chemotherapeutic drug-induced intrinsic apoptosis by a mechanism that critically depends on caspase-2.

### 3.5. Increased TRIM25 Binding to 5′UTR of Caspase-2 by Doxorubicin

**Figure 8:**
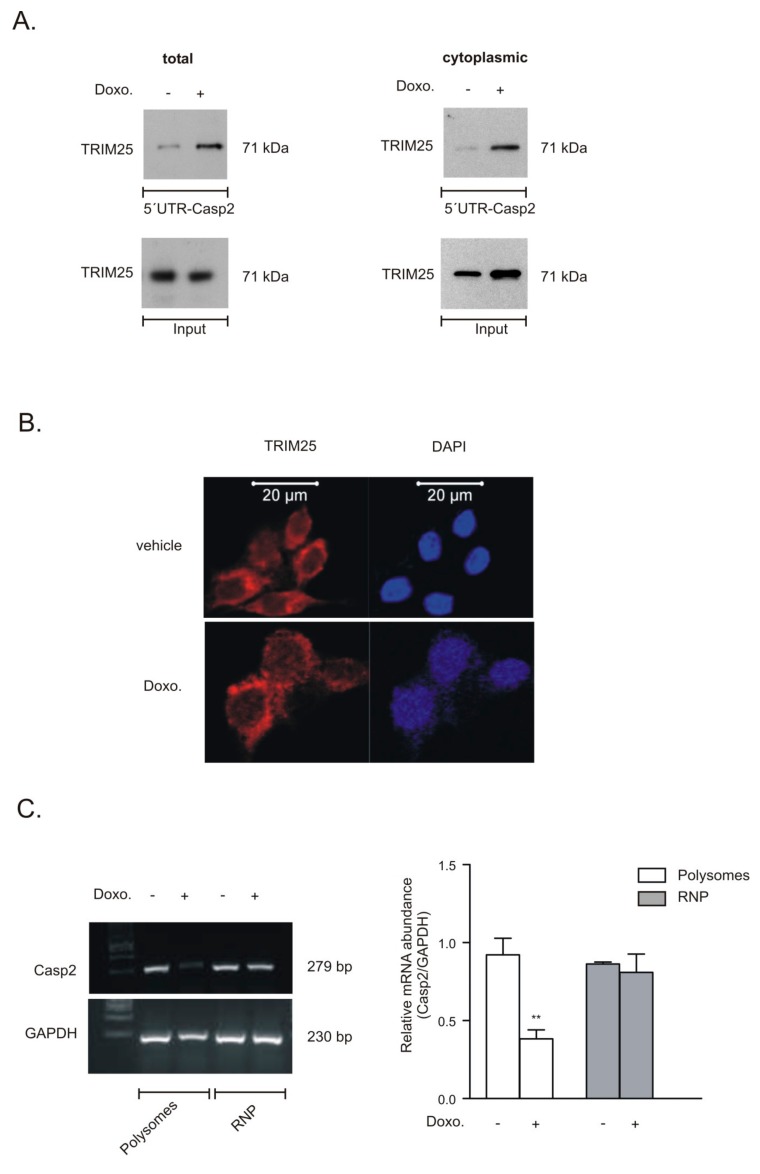
Doxorubicin induces TRIM25 binding to the 5′UTR of caspase-2 and reduces recruitment of caspase-2 mRNA at polysomes. (**A**). Representative biotin pull-down assays showing doxorubicin-induced TRIM25 affinity to the 5′UTR of caspase-2. The biotinylated transcript, which encompasses 241 nucleotides of the 5′UTR of the human caspase-2 (5′UTR-Casp2), was incubated with either total (left) or cytoplasmic (right) cell lysates from vehicle-treated DLD-1 cells (−) or from cells treated with 10 µg/mL of doxorubicin (+) for 6 h. RNA binding of TRIM25 to the pull-down material was assessed by Western blot analysis and an equal input TRIM25 levels were assayed by Western blot analysis (input). Blots shown are representative of two independent experiments with similar results. (**B**). DLD-1 cells were stimulated for 6 h with either vehicle or with 10 µg/mL doxorubicin (Doxo.) before intracellular TRIM25 was analyzed by confocal microscopy. Cell nuclei were visualized by DAPI staining (blue panel). Bar: 20 µm. (**C**). DLD-1 cells were stimulated for 6 h either with vehicle (−) or doxorubicin (+) before fractions of translational inactive RNPs were separated from translational active polysomes by ultracentrifugation. The amount of caspase-2 mRNA and GAPDH mRNA was determined by semiquantitative RT-PCR using primer pairs that were complementary and specific to the coding region of both genes The graph on the right panel shows means ± SD (*n* = 3) and depicts the amount of caspase-2 mRNA levels normalized to GAPDH mRNA in polysomes (open bars) relative to those in RNP (grey bars) fractions. ** *p* ≤ 0.01, doxorubicin vs. vehicle-treated cells.

Next, we investigated whether treatment of cells with chemotherapeutic drugs would affect the constitutive binding of TRIM25 to the 5′UTR of caspase-2 mRNA. For this purpose, we employed a streptavidin-tethered pull-down assay with an in vitro-transcribed biotinylated mRNA encompassing the complete 5′UTR of caspase-2. Since only doxorubicin was able to significantly enhance activation of effector caspases in DLD-1 as well as in RKO cells, the following experiments were performed with this drug. Exposure of DLD-1 cells with doxorubicin caused a clear increase in TRIM25 affinity to the 5′UTR of caspase-2 (Figure 8A) and similar results were obtained with RKO cells (data not shown). Furthermore, the fact that the drug-evoked increase in TRIM25-caspase-2 RNA binding was found in total and in cytoplasmic cell lysates indicates that the interaction mainly occurs in the cytoplasm. By employing confocal microscopy, we observed that TRIM25 is mainly localized in the cytoplasm and the pattern of TRIM25 staining was not affected after cells were treated with doxorubicin (Figure 8B). In contrast, there was no staining if the primary antibody was omitted, indicating that the immune-positive signals were specific (data not shown). Finally, we tested whether the doxorubicin-induced increase in TRIM25 binding to the 5′UTR-caspase-2 corresponded with a reduced translation of caspase-2. To this end, we monitored drug-induced changes in the ribosome occupancy of caspase-2 mRNA by polysome/RNP fractionation experiments using sucrose density gradient centrifugation. As previously demonstrated, long-term treatment of DLD-1 cells with doxorubicin at the concentration routinely used induces a global inhibition in translation [8]. For this reason, we chose a short incubation period of 6 h. Doxorubicin significantly reduced the basal content of caspase-2 mRNA in polysomes when compared with GAPDH mRNA but had no effect on the contents of corresponding mRNAs associated with RNPs (Figure 8C), thus implicating that caspase-2 translation is reduced by doxorubicin. Together, these data demonstrate that transient siRNA-dependent depletion of TRIM25 via increased caspase-2 translation sensitizes colon carcinoma cells to the intrinsic apoptotic pathway.

## 4. Discussion

In this study, we reported on the identification of TRIM25 as a novel potential regulator of caspase-2 translation in human colon carcinoma cells. Despite most of the originally described functions by TRIM25 being assigned to its E3 ubiquitin ligase activity, recent data from cross-linking immunoprecipitation high-throughput sequencing (CLIP-seq) or related approaches indicate that TRIM25 and some other members of the TRIM family of E3 ligases can influence the processing and stability of bound mRNA targets due to specific RNA-binding capacities [33] (for a review, see [25]). Using RNA-affinity chromatography, we demonstrated that TRIM25 is able to bind the 5′UTR of caspase-2 (Figure 1). Importantly, in the same experiment, the mRNA-binding protein human antigen R (HuR), which we previously identified as a novel negative regulator of internal ribosome entry site (IRES)-triggered caspase-2 translation [6,8], was identified to associate with the 5′UTR of caspase-2 with a similar affinity, which underpins the validity of our experimental approach for identification of caspase-2 mRNA-binding proteins (Supplementary Material). A specific binding of TRIM25 to caspase-2 mRNA in both cell lines was furthermore confirmed by RNA-pulldown analysis and RIP assays using TRIM25-specific antibodies (Figure 1). Although none of the TRIM family members contain any typical RNA-binding domains (RBP), the high RNA-binding affinity of TRIM25 is structurally mainly attributed to the C-terminal PRY/SPRY domain of the protein [34,35] while the coiled coil (CC) domain, which is critical for dimerization, seems indirectly relevant for the RNA-protein interaction [33]. The key finding of our study is that inhibition of caspase-2 by TRIM25 constitutes a so far unrecognized pathway, which may be relevant for the resistance of human colon carcinoma cells to chemotherapy-induced apoptosis. Specifically, by using siRNA-mediated depletion and overexpression of human TRIM25, we demonstrated that TRIM25, via binding to the 5′UTR, does negatively interfere with the translation of caspase-2. Importantly, in clear contrast to RKO cells, overexpression of TRIM25 in HEK cells had no comparable effects on caspase-2 levels (Figure 2B), suggesting that the negative regulation of caspase-2 by TRIM25 could probably reflect a tumor-specific phenomenon. It is tempting to speculate that TRIM25, in addition to its reported degradative activity on let-7 precursors [36], may stabilize certain tumor cell-specific miRNAs. To the best of our knowledge, this is the first report demonstrating a translation modulatory activity by TRIM25. This assumption is corroborated by the following observations. Firstly, the TRIM25 depletion-dependent increase in caspase-2 is exclusively on the protein level but not on the mRNA level, thus excluding the possibility that TRIM25 may negatively affect caspase-2 transcription or the stability of caspase-2 transcripts. Furthermore, the TRIM25 depletion-dependent increase in caspase-2 is completely blunted by cycloheximide (Figure 4), indicating that TRIM25 mainly interferes with de novo synthesis of caspase-2. Regulation at the level of protein synthesis is further underlined by the finding that silencing of TRIM25 caused a significant shift in caspase-2 mRNA allocation from polysomes to translationally inactive RNP fractions when compared with the caspase-2 distribution in control siRNA-transfected cells (Figure 4). Together, these results indicate that TRIM25, in addition to the reported effects on transcriptional and posttranscriptional gene expression [26,33], can directly interfere with the translation of mRNAs. However, the underlying mechanisms are still unknown. Potentially, the binding of TRIM25 to the 5′UTR of caspase-2 may lead to the ubiquitination of neighboring RNA-binding proteins or translation regulatory factors critically involved in cap-dependent or IRES-mediated translation; the latter one seems critical for the control of caspase-2 in colon carcinoma cells [8]. In addition, TRIM25 may utilize caspase-2 mRNA as a scaffold to target translation initiation or elongation factors for proteasome-dependent degradation, which may further implicate direct mRNA regulation by the ubiquitin proteasome system. Alternatively, TRIM25 could act as a negative IRES trans-acting factor (ITAF) independent of its ligase activity, which sterically hinders the recruitment of the 40S ribosome. Further investigations are needed to unveil the detailed mechanism underlying inhibition of caspase-2 translation by TRIM25.

In accordance with this study, we previously demonstrated that the mRNA-binding protein HuR through constitutive binding to the 5′UTR of caspase-2 mRNA exerts a direct inhibitory effect on caspase-2 translation, a mechanism that critically contributes to the therapy resistance of human colon carcinoma cells [6,7,8]. Here, we demonstrated a significantly elevated sensitivity towards intrinsic apoptosis induced by topoisomerase inhibitors (doxorubicin and etoposide), as indicated by the increased cleavage of effector caspase-3 (Figure 5). The finding that cells were rescued from TRIM25 depletion-dependent apoptosis after additional knockdown of caspase-2 underlines the critical role of caspase-2 for apoptosis-sensitizing effects by TRIM25 silencing (Figure 7). Interestingly, the upregulation of caspase-2 upon TRIM25 silencing alone is unable to trigger apoptosis in colon carcinoma cells but requires an additional apoptosis-inducible stimulus, such as doxorubicin. This is fully compatible with the assigned role of caspase-2 to mainly act as a damage sensor, allowing cells to initiate apoptosis as the last resort to genotoxic insults [11,37]. Consequently, drug-induced TRIM25 binding, which is accompanied by a reduced translation of caspase-2, may constitute a novel cell survival mechanism by TRIM25. In accordance with our data, a previous study demonstrated that TRIM25 can dampen p53-dependent DNA damage-induced cell death in the human colon carcinoma cell line HCT116 via inhibition of p53 ubiquitination and degradation [38]. These data suggest that in colon carcinoma cells, TRIM25 can exert a broad anti-apoptotic program through diverse mechanisms. Interestingly, overexpression of TRIM25 has been reported in several human cancers, including breast [26], ovarian [39], prostate [34], gastric [40], and lung [41]. In addition, TRIM25 via induction of TGFβ signaling pathways promotes proliferation and invasion of colorectal cancer cells [42]. Consistently, TRIM25 is significantly upregulated in colorectal cancer tissues and different human colorectal cancer cell lines [42], thus implying a tumorigenic role of TRIM25 in these carcinomas. Together, our study implicates that TRIM25-dependent inhibition of caspase-2 may represent a so far unrecognized survival mechanism of colorectal carcinoma cells. Targeting TRIM25 may reflect a novel therapeutic avenue to improve the efficacy of current therapy regimes for the treatment of CRC.

## 5. Conclusions

Together, our study identified the E3 ligase TRIM25 as a novel caspase-2 RNA-binding protein in colon carcinoma cells, which negatively affects protein expression of caspase-2. Our finding indicates that besides the described roles in regulating the stability of pre-let-7a [36] and the postulated functions in suppressing gene expression at the transcriptional and/or post-transcriptional levels [26], TRIM25 may directly interfere with mRNA translation. Functionally, the herein described TRIM25-dependent inhibition of caspase-2 could represent a so far unrecognized post-transcriptional survival mechanism utilized by colorectal carcinoma cells, and further supports the tumorigenic capacity of this particular TRIM member. With regard to the multifunctional roles of caspase-2 in apoptosis and diverse non-apoptotic processes, the negative regulation of caspase-2 by TRIM25 may have a strong impact on cell survival and drug resistance. Targeting TRIM25 may therefore reflect an attractive therapeutic avenue to improve the efficacy of current therapy regimes for the treatment of CRC.

## Supplementary Materials

The supplementary figures and table are available online at https://www.mdpi.com/2073-4409/8/12/1622/s1, Figure S1: Time-dependent changes in caspase-2 protein levels after TRIM25 knockdown in DLD-1 and RKO cells, Figure S2: Time-course of steady-state levels of TRIM25 and caspase-2 mRNA after transient RNAi-mediated TRIM25 knockdown in DLD-1 and RKO cells, Figure S3: DLD-1 were transfected with control siRNA duplexes (siCtrl.) or with siRNA duplexes of TRIM25 (siTRIM25) for 24 h before translation was blocked by the addition of cycloheximide, Table S1: List of caspase-2 mRNA-binding proteins (depicted by gene names) as identified by LC/MS using the biotin-labeled in vitro-transcribed 5′UTR and coding region (CDR) of caspase-2 as a bait.
