# Supplementary material for: Identification of TRIM25 as a Negative Regulator of Caspase-2 Expression Reveals a Novel Target for Sensitizing Colon Carcinoma Cells to Intrinsic Apoptosis
Source: Cells. 2019 Dec 12;8(12):1622. doi: 10.3390/cells8121622 (PMC6952940; doi:10.3390/cells8121622)
Supplement: Supplementary file 1 [file cells-08-01622-s001.pdf]

cells MDPI

Supplementary Material

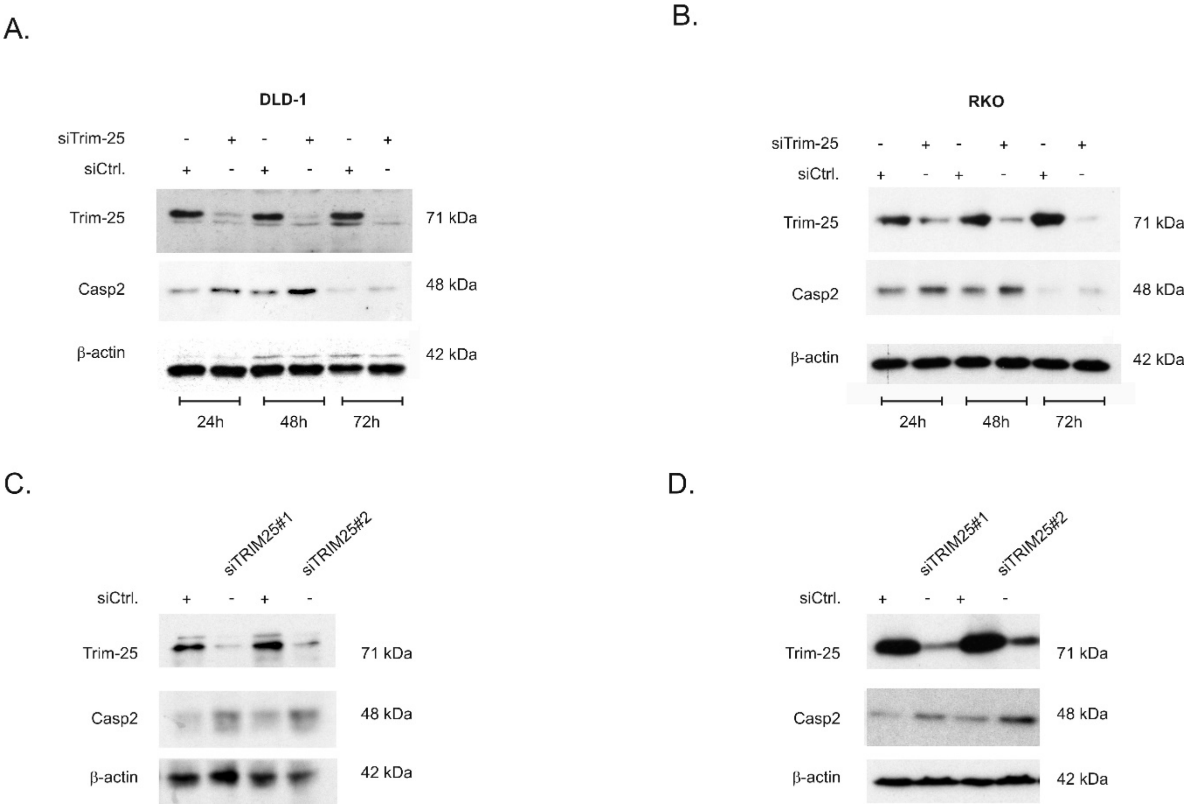

Supplementary Figure 1. (**A, B**). Time-dependent changes in caspase-2 protein levels after TRIM25 knockdown in DLD-1 (**A**) and in RKO (**B**) cells. Subconfluent colon carcinoma cells were transfected with control siRNA duplexes (siCtrl.) or with siRNA duplexes of TRIM25 (siTRIM25) for the indicated time periods before the content of total TRIM25 or caspase-2 (Casp2) was monitored by Western blot analysis, with β-actin used as a loading control. For Western blotting, equal amounts of protein (20 µg) from total cell homogenates were subjected to SDS-PAGE and probed with the corresponding antibodies using β-actin as a loading control. Data shown are representative for three independent experiments giving similar results. (**C, D**). The increased levels of caspase-2 by TRIM25 knockdown in DLD-1 (**C**) and RKO (**D**) cells were analyzed 48 h after siRNA transfection and validated with two different sets of siRNA duplexes complementary to distinct regions of the TRIM25 mRNA (siTRIM25#1, siTRIM25#2) as described in the materials and methods.

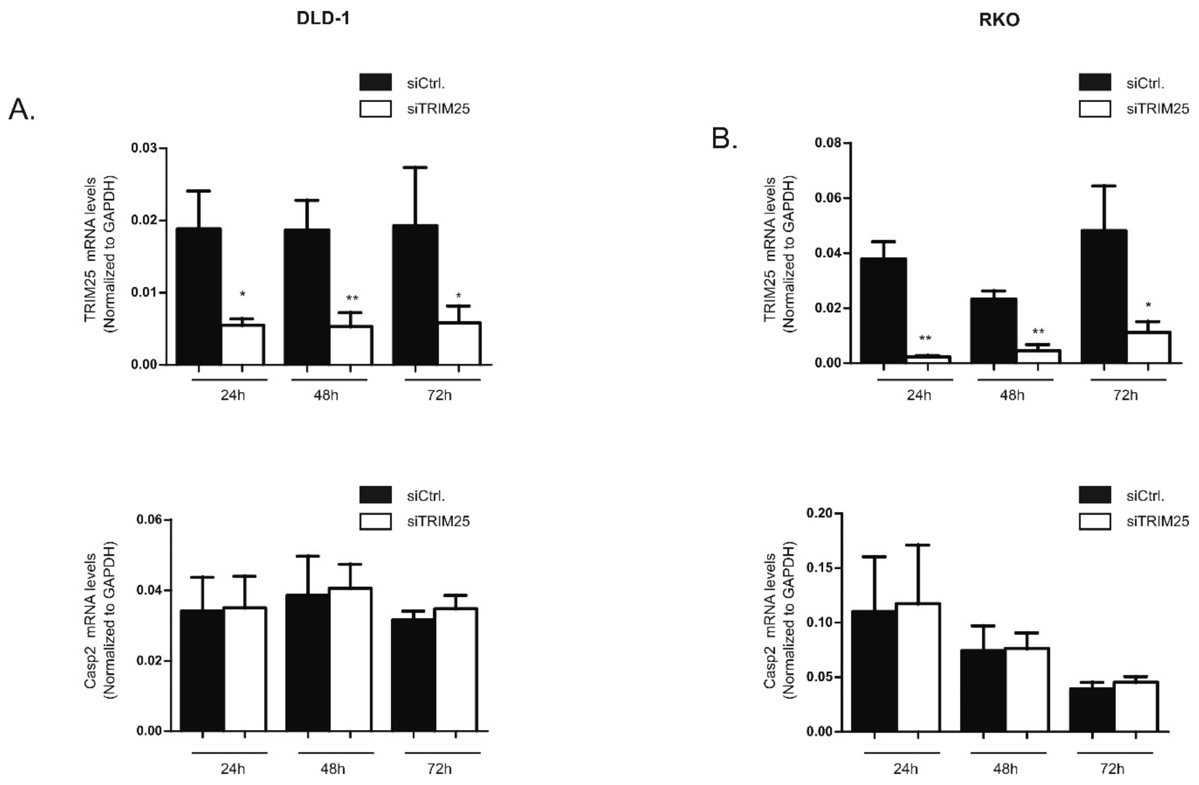

Supplementary Figure 2. Time course of steady-state levels of TRIM25 (upper panels) and caspase-2 (lower panels) mRNA after transient RNAi-mediated TRIM25 knockdown. DLD-1 (**A**) or RKO (**B**) cells were transiently transfected with control duplexes (siCtrl.) or with siRNA duplexes of TRIM25 (siTRIM25) for the indicated time periods before cells were harvested and extracted for total cellular RNAs. Changes in steady-state TRIM25 mRNA (black bars) and caspase-2 mRNA levels (white bars) were measured by quantitative real-time PCR in relation to GAPDH steady-state mRNA levels. Data represent means ± SD (n = 3), * $p \leq 0.05$, ** $p \leq 0.01$ siCtrl. cells vs. siTRIM25-transfected cells.

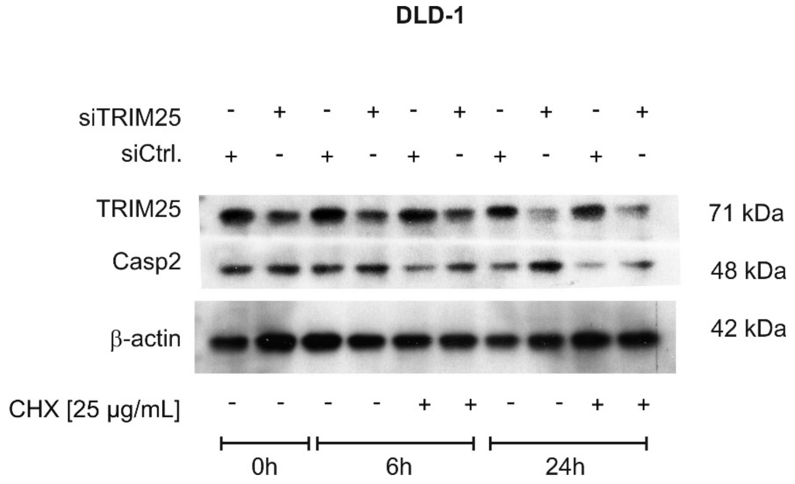

Supplementary Figure 3. DLD-1 were transfected with control siRNA duplexes (siCtrl.) or with siRNA duplexes of TRIM25 (siTRIM25) for 24 h before translation was blocked by the addition of cycloheximide (CHX, 25 μg/mL) (0 h). After the indicated time periods, cells were harvested and extracted for total protein lysates. For Western blotting, equal amounts of protein (20 μg) from total cell homogenates were subjected to SDS-PAGE and probed with the corresponding antibodies using β-actin as a loading control. Data shown are representative of two independent experiments giving similar results.
